# The relationship between sitting height, sitting height to height ratio with blood pressure among Polokwane private school children aged 6–13 years

**DOI:** 10.1186/s12889-017-4983-3

**Published:** 2018-01-04

**Authors:** Nthai E. Ramoshaba, Kotsedi D. Monyeki, Joyce Mpya, Mafolwa S. Monyeki

**Affiliations:** Department of Physiology & Environmental Health, University of Limpopo, Polokwane, South Africa

**Keywords:** Sitting height, Blood pressure, Private school, Children, South Africa

## Abstract

**Abstract:**

**Background:**

It is notable that sitting height (SH) correlates with blood pressure (BP) in children and adolescents of developed countries. However, little is known about the relationships between SH and SH to height ratio (SH/H) with BP in South African children from middle and upper socio-economic groups. The purpose of this study was to compare SH and SH/H of private school attending children in the Polokwane area with National Health and Nutrition Examination Survey III (NHANES III) reference population and to determine the relationship between SH, SH/H with BP among private school attending children.

**Methods:**

A total of 1665 children (846 boys and 819 girls) aged between 6 and 13 years attending three private schools in Polokwane, underwent anthropometric and BP measurements using standard procedures. Linear regression was used to determine the relationship between height, SH, SH/H with BP among these children.

**Results:**

Polokwane private school attending boys from age 7 to 13 years displayed a lower mean SH compared to the NHANES III whereas NHANHES III girls from age 10 to 13 years had a higher mean SH compared to those in private school. In the simple regression analysis, SH was positively associated with SBP (β =1.318; 95% CI = 1.217–1.418) and DBP (β = 0.641; 95% Cl = 0.555–0.727). The findings remains statistically significant only for SH with both SBP (β = 1.025; 95% Cl = 0.844–1.201) and DBP (β = 0.585; 95% Cl 0.434–0.736) after adjusting for age, gender and BMI among these children.

**Conclusion:**

In South African children, SH and SH/H were lower compared to the NHANES III children. There was a significant positive association between DBP and SBP together with the components of height among Polokwane private school children.

## Background

High blood pressure (BP) is a major public health obstacle that leads to morbidity and mortality in both developed and developing countries [1–3]. Existing literature indicates that sustained high BP in children is associated with the risk factors of cardiovascular diseases (CVD) in sub-Saharan African adults [4]. Moreover, child growth proportion has shown close links with the development of CVD risk factors. Most studies reported that height can be used as a predictor of high BP in children and adolescents [5–7].

Height can be divided into three components, sitting height (SH), leg length and SH to height ratio (SH/H) which are pivotal in the assessment of growth proportion. SH not SH/H was reported to be associated with BP in Brazilian children [8]. Furthermore, Dong et al. [9] study exhibited that SH was associated with BP in Chinese children and adolescents. Recent findings showed that significant relationships between SH with both systolic BP (SBP) and diastolic BP (DBP) in rural South African children exists [10].

The association between SH and SH/H with BP in children from middle and upper socio-economic status with the prevalence of hypertension ranged from 0.9–12.9% and overweight ranged from 0 to 22.6% is scant [11]. To reiterate, the purpose of the study was first, to compare SH and SH/H of Polokwane private school attending children with the National Health and Nutrition Examination Survey III (NHANES III) reference population. Finally, it was to determine the relationship between SH and SH/H with BP among Polokwane private school attending children.

## Methods

### Study population and ethical approval

A total of 1665 children (846 boys and 819 girls), aged 6 to 13 years, attending three private schools in Polokwane, a city in the Limpopo Province of South Africa, participated in the study. The majority of participants were black children (99.77%), while 0.2% were white and coloureds made up the 0.01% with Indians at 0.02% were in a minority and as a result were excluded in the analysis. Generally, children attending private schools in South Africa fall within the middle and upper socio-economic groups of the population. All the children who were present at the schools during the days of the survey participated in the study. The Ethics Committee of the University of Limpopo granted ethical approval prior to the study. Written informed consent was obtained from parents or guardians.

### Anthropometric measurements

The children were anthropometrically measured using the International Society for the Advancement of Kinanthropometry (ISAK) [12]. A Martin anthropometer was used to measure height to the nearest 0.1 cm. The SH was measured by bringing the horizontal bar of Martin anthropometer to the most superior midline of the head while the child was sitting in the erect position on a flat stool or box. Weight was measured on an electronic scale to the nearest 0.1 kg. Body mass index (BMI) was calculated as weight (kg)/ (height (cm)) ^2^.

### Blood pressure

Using an electronic Micronta monitoring kit (Omron), at least three BP readings of SBP and DBP were taken at 5 min intervals after the child had been seated for 5 min or longer [13, 14]. The bladder device contains an electronic infrasonic transducer that monitors the BP and pulse rate, displaying those concurrently on the screen. This versatile instrument has been designed for research and clinical purposes. In a pilot study, conducted before the survey, a high correlation (*r* = 0.93) was found between the readings taken with the automated device and those taken with a conventional Sphygmomanometer.

### Statistical analysis

Descriptive statistics were performed for height, SH, SH/H, and BP with the Polokwane private school children aged 6–13 years. Student’s t-test was utilised to test the significant difference between the genders. SH and SH/H of private school children were compared with NHANES III reference population [15]. The linear regression models were used to analyse the relationship between BP (SBP: Systolic Blood Pressure and DBP: Diastolic Blood Pressure) and components of height (height, SH and SH/H) and were unadjusted and adjusted regarding to age and gender. All statistical analyses were performed using the Statistical Package for the Social Sciences (SPSS) version 23. The statistical significance was set at *P* < 0.05.

## Results

**Fig. 1:**
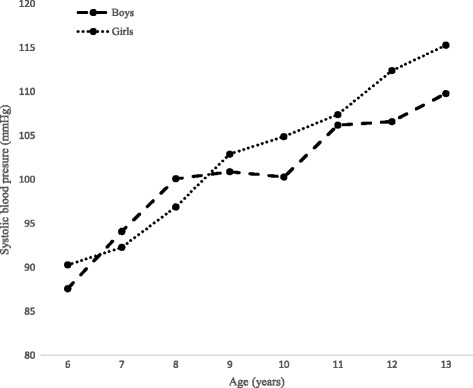
The comparison of systolic blood pressure by age among Polokwane private schools children

**Fig. 2:**
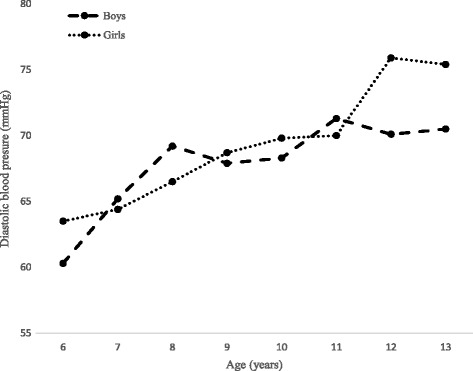
The comparison of diastolic blood pressure by age among Polokwane private school children

Table 1 reflects descriptive statistics for height, SH and SH/H among Polokwane private school boys and girls aged 6–13 years. At age 11–13 years, girls mean SH (76.5 cm - 81.3 cm) were significantly (P < 0.05) higher than boys mean SH (74.4 cm - 78.9 cm). Overall, girls’ mean SH (72.0 cm) was significantly higher than boys mean SH (71.8 cm). Both SBP and DBP increased proportionally with age for both genders (Figs. 1 and 2).

**Table 1:** Descriptive statistics for height, sitting height and sitting height/height ratio of Polokwane private schools children, age 6–13 years

	Sample size		Height (cm)		SH (cm)		SH/H (%)	
Age (years)	Boys	Girls	Boys	Girls	Boys	Girls	Boys	Girls
			M (SD)	M (SD)	M (SD)	M (SD)	M (SD)	M (SD)
6	74	94	122.8 (5.4)	121.6 (5.8)	65.7 (2.9)	65.1 (2.7)	53.5 (1.2)	53.6 (1.7)
7	118	122	126.8 (6.0)	126.2 (7.5)	67.0* (3.2)	66.2* (2.8)	52.9 (1.8)	52.5 (2.1)
8	116	117	131.5 (6.9)	131.7 (5.2)	68.6 (3.0)	68.6 (2.5)	52.3 (1.9)	52.1 (1.3)
9	106	106	136.1 (6.9)	137.5 (6.6)	70.5 (3.2)	71.2 (3.4)	51.8 (1.6)	51.8 (1.5)
10	117	104	142.1* (6.0)	144.0* (6.8)	72.8 (2.8)	73.6 (5.3)	51.3 (1.4)	51.1 (1.8)
11	140	140	146.6* (6.6)	150.0* (7.7)	74.4* (3.1)	76.5* (3.7)	50.8 (1.7)	51.0 (1.3)
12	104	93	151.2* (8.2)	155.7* (7.8)	76.7* (3.4)	79.0* (3.5)	50.7 (1.3)	50.8 (1.5)
13	71	43	156.3* (7.3)	160.0* (7.0)	78.9* (3.6)	81.3* (2.8)	50.5 (1.7)	50.9 (1.7)
Total	846	819	139.2* (12.3)	139.4* (13.9)	71.8* (5.2)	72.0* (6.0)	51.7 (0.12)	51.8 (0.12)

*= *P* < 0.05; *M* mean, *SD* standard deviation, *SH* sitting height, *SH/H* sitting height/height ratio

**Fig. 3:**
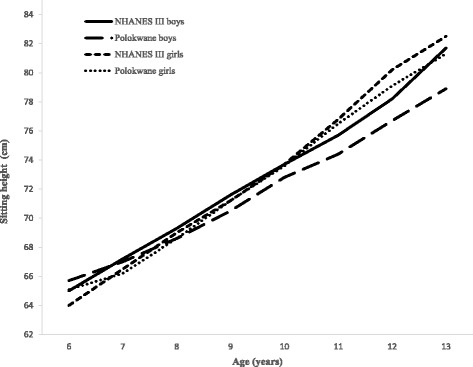
The comparison of mean sitting height between Polokwane and NHANES III children

**Fig. 4:**
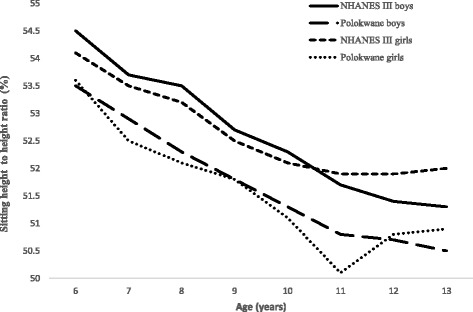
The comparison of mean sitting height to height ratio between Polokwane and NHANES III children

Figs. 3 and 4 showed the comparison between SH, SH/H mean values between Polokwane private school and NHANES III children aged 6–13 years. Polokwane private school boys showed a lower mean SH compared to NHANES III boys from age 7 to 13 years whereas NHANHES III girls had a higher mean SH compared to private school girls from age 10 to 13 years. Figure 4 exhibits an inverse decrease in SH/H with age, where mean SH/H among Polokwane private school children were low compared to the NHANES III children for both genders.

Table 2 showed linear regression for the association between height, SH, SH/H with BP in Polokwane private school attending children, unadjusted and adjusted for age, gender and BMI. There was a significant association (*P* < 0.001) between SH with SBP (β = 1.318; 95% CI = 1.217–1.418) and DBP (β = 0.641; 95% Cl = 0.555–0.727) for unadjusted. After adjusted for age, gender and BMI, SH was significantly associated with both SBP (β = 0.691; 95% Cl = 0.509–0.872) and DBP (β = 0.304; 95% Cl 0.148–0.460) among these children. Furthermore, SH/H was associated (*P* < 0.05) with SBP and DBP for unadjusted only.

**Table 2:** Linear regression coefficients (β), *P*-value and 95% confidence intervals for the association between sitting height (SH), SH to height ratio (SH/H) and blood pressure among participants

	Unadjusted				Adjusted for age, gender and BMI			
	Coefficient (β)	95% Cl		*P*-value	Coefficient (β)	95% Cl		*P*-value
SBP (mmHg)								
Height (cm)	0.540	0.221	0.387	< 0.001	0.304	0.221	0.387	< 0.001
SH (cm)	1.318	1.217	1.418	< 0.001	0.691	0.509	0.872	< 0.001
SH/H	−142.147	−177.302	−106.991	< 0.001	−16.336	−51.482	18.810	< 0.362
DBP (mmHg)								
Height (cm)	0.254	0.217	0.291	< 0.001	0.139	0.069	0.216	< 0.001
SH (cm)	0.641	0.555	0.727	< 0.001	0.304	0.148	0.460	< 0.001
SH/H	−53.111	−80.461	−25.760	< 0.001	−9.876	−39.692	19.940	0.516

*CI* confidence interval, *SBP* systolic blood pressure, *DBP* diastolic blood pressure, *BMI* body mass index

## Discussion

This cross-sectional study described the relationship between SH, SH/H and BP among children aged between 6 to 13 years attending private school in Polokwane. SH and SH/H was significantly associated with SBP and DBP in the unadjusted analysis. However, after being adjusted for age, gender and BMI, only SH remained associated with both SBP and DBP.

An increase in age of children increases with the mean SH and decreases with the mean SH/H in the current study. This is in agreement with others studies reported that height increases with age and is mainly due to an increase in lower limb rather than in upper limb or SH [16–20]. However, girls attending private schools in Polokwane showed a significantly high mean SH compared to that of boys in similar schools. This could be due to the fast growth rate of girls compared with that of boys between 10 and 13 years of age as reported by Alan et al. [21]. Furthermore, an increase in mean SH and decreasing in mean SH/H of NHANES III girls and boys with an increase in age was observed. However, SH and SH/H values of NHANES III boys and girls were higher compared to their counterparts, private school attending boys and girls in Polokwane (Fig. 3-4). Indicating that in the pre-pubertal years, growth occurred more in the lower limbs than in the trunk in private school children as compared to NHANES III children. Moreover, this may be due to that private school attending children are/were from middle and upper socio-economic backgrounds in South Africa [22] while the NHANES III children were from developed countries.

Current results showed that SH is positively associated with SBP and DBP for both unadjusted and adjusted for age, gender and BMI among Polokwane private school attending children. Similar findings were reported among Chinese [9], Brazilian [8] and rural South African [10] children and adolescents. The possible explanation for these findings could be that BP at the heart level must surpass the hydrostatic pressure induced by vertical distance between the heart and the head, to ensure an adequate perfusion of a child’s brain [23].

Based on a large sample in this study, the recognition of a significant association between components of height and BP in children and adolescents was examined for the first time in Polokwane private schools, South Africa. Components of height are simple, make use of inexpensive tools which can be used as an indicator of high BP among children [10, 23]. Furthermore, BP and components of height are more advantageous, due to their applicability and they are easily understood by the literate and illiterate population, particularly in areas of South Africa.

This study did not consider detailed information such as socio-economic and nutritional status of the children’s families, dietary intake, physical activity and family history of hypertension. The BP and anthropometric measurements were taken directly; hence, recall or estimation bias will not prevail in our study. In addition, we measured BP during early childhood, signifying that early monitoring should commence from children’s early days in order to identify and screen individuals that show vulnerability to associated risk factors [24].

## Conclusion

In South African children, SH and SH/H were low compared to the NHANES III children. There was significant association between SH with SBP and DBP, when unadjusted and adjusted for age, gender and BMI among the children attending private schools in Polokwane. In addition, SH can be used as a predictor of high BP among these children. Further interrogation of the relationship between components of height and BP in South African children is recommended overtime in order to shed more light on disease development and the factors thereof.

## Abbreviations

BMI: Body mass index
BP: Blood pressure
DBP: Diastolic blood pressure
ISAK: International Society for the Advancement of Kinanthropometry
NHANES III: National Health and Nutrition Examination Survey III
SBP: Systolic blood pressure
SH: Sitting height
SH/H: Sitting height to height ratio
